# Machine learning based shear strength prediction in reinforced concrete beams using Levy flight enhanced decision trees

**DOI:** 10.1038/s41598-025-12359-y

**Published:** 2025-07-28

**Authors:** Aybike Özyüksel Çiftçioğlu, Anıl Delikanlı, Torkan Shafighfard, Faramarz Bagherzadeh

**Affiliations:** 1 Department of Civil Engineering, Faculty of Engineering and Natural Sciences, Manisa Celal Bayar University, Manisa, Turkey; 2 Campbell Terrace, Petone, Lower Hutt, New Zealand; 3 Faculty of Mathematics and Computer Science, University of Bremen, Bremen, Germany

**Keywords:** Hybrid machine learning framework, Interpretable artificial intelligence, Non-linear shear capacity modeling, Reinforced concrete T-Beams, Stochastic optimization, Mechanical properties, Civil engineering

## Abstract

Reinforced concrete (RC) T-beams are widely used in structural systems due to their efficient geometry and load-carrying capacity. However, accurately predicting their shear strength remains a challenge, particularly under complex loading scenarios. Conventional empirical approaches often struggle to adequately represent the complex and nonlinear relationships among structural design variables. In this study, a novel machine learning approach, termed Levy-DT, is introduced to enhance the prediction accuracy of shear strength in RC T-beams. The proposed method combines the structure of Decision Tree algorithm with Levy Flight, a stochastic optimization technique, to improve global search capabilities and avoid local minima. A comprehensive dataset comprising 195 experimentally tested T-beams is used to train and evaluate six different regression models, including optimized Decision Tree, Random Forest, AdaBoost, K-Nearest Neighbors, Ridge Regression, and the proposed Levy-DT. Model performance is assessed using multiple metrics such as R², RMSE, and MAE, with cross-validation employed for robustness. Systematic hyperparameter optimization is implemented for the baseline Decision Tree to ensure fair comparison. The results show that Levy-DT outperforms all other models, achieving the highest prediction accuracy with strong generalization. To further understand the model’s decision-making process, SHAP analysis is carried out, identifying axial force and reinforcement depth as key contributors to the shear strength estimation. This study highlights the potential of integrating optimization techniques with machine learning for reliable and interpretable structural predictions.

## Introduction

Reinforced concrete (RC) T-beams are essential elements in many load-bearing systems within civil engineering due to their dependable structural performance under various service conditions^1^. These beams possess a distinct T-shaped profile, where a horizontal flange is integrated with a vertical web, a configuration engineered to enhance both stiffness and load transfer efficiency^2^. Such a design proves especially effective in components like floor slabs and bridge decks, where simultaneous resistance to flexural and shear forces is required. The flange not only improves flexural capacity but also allows for load distribution over extended spans, promoting continuity and structural integrity. Consequently, RC T-beams are frequently employed in both conventional and modern structural systems. Their ability to be tailored to specific architectural and mechanical demands enhances their applicability across diverse structural layouts^3^, supporting efficient material use and steady performance under varied loading environments. Moreover, the inherent strength and long-term durability of RC make these beams suitable for conditions involving high loads or harsh exposures. Still, capturing their structural response particularly under combined effects such as axial load, bending, and shear remains an intricate challenge. Among these, shear behavior demands particular attention, as it directly affects both serviceability and failure risk^4,5^.

In design, the shear strength of RC T-beams becomes especially important when these members experience significant interaction between shear and flexure forces^6,7^. An accurate estimation of shear capacity is vital to prevent brittle failures, which often occur suddenly and can threaten structural integrity^8,9^. Ensuring that this capacity is properly evaluated is essential for safe design under routine service and extreme loading conditions. Despite the widespread use of empirical and semi-theoretical models, these conventional methods often fall short when applied across a broad range of T-beam geometries and loading patterns. Most of them are based on some assumed conditions that do not necessarily reflect the true complexities of actual behavior in structures. Variations in concrete strength and reinforcement specifications, the shape of the beam, and loading conditions cannot be fitted to the uncertainties of traditional design equations. In addition, conventional design approaches are based on safety factors that do not seem to reflect the true shear behavior of RC T-beams in all situations. Consequently, the accuracy of such applications can be low with regularly shaped members or normal material properties. This limitation calls for an upsurge in demand for newer and more credible approaches to assess the shear strength of RC T-beams with reasonable accuracy. Despite decades of investigation, estimating the shear strength of RC T-beams with sufficient accuracy continues to pose a significant challenge in structural engineering^7^. This difficulty largely stems from the inherently nonlinear and complex nature of shear behavior, where several mechanisms such as the progression of concrete cracking, the interaction of coarse aggregate particles, dowel action of longitudinal reinforcement, and the contribution of transverse reinforcement operate concurrently and are highly interdependent^10^. These mechanisms are influenced by multiple variables, including cross-sectional dimensions, reinforcement configuration, type and application point of loading, and variations in material properties. Additionally, the stress field near the junction of the web and flange in T-beams is often discontinuous, creating a divergence from the assumptions of classical beam theory and further complicating analytical modeling^7^. Most empirical formulations embedded in current design standards are calibrated primarily for rectangular cross-sections and do not fully account for the geometric and mechanical complexities introduced by flanges. These traditional approaches are generally developed from experimental findings or simplified theoretical insights. Despite their advantages, RC T-beams are often limited by prediction inconsistencies across varying design scenarios. A comprehensive theoretical model capable of universally describing shear behavior has yet to be established. Experimental findings are often impacted by discrepancies in specimen geometry, loading arrangements, and support conditions, leading to variable outcomes^11^. These complexities highlight the need for adaptable modeling tools that can account for a wide spectrum of influencing parameters^12–21^. In response to these limitations, data-driven approaches especially those grounded in machine learning (ML) have gained traction within the structural engineering community. By uncovering hidden relationships and modeling nonlinear behaviors, ML techniques frequently surpass conventional analytical models in predicting shear strength^22–24^.

In domains where classical theories fall short, ML has been widely adopted to model intricate systems^25,26^. In civil engineering specifically, ML methods have demonstrated considerable promise, particularly in problems involving multiple interrelated factors and nonlinearities^27–30^. Their capacity to handle complex datasets and extract meaningful patterns makes them highly suitable for simulating the behavior of elements such as RC T-beams^31–35^. Within structural analysis, where numerous parameters interact, the strength of ML lies in its adaptability and pattern recognition capabilities. These algorithms can effectively capture and represent such complex nonlinear interactions under varying conditions with changes in material properties, geometric configuration, and force application. The attributes of ML thus provide an appealing alternative to the traditional empirical and analytical techniques, which usually base their approach on simplified assumptions that often fail to accurately capture the complexity of real-world structural behavior^36–40^. Algorithms and computing capability enable ML models to perform examinations on vast amounts of data to describe intricate patterns and relationships overlooked by older analytical approaches^41–44^. This is especially critical in structural engineering, where the performance of elements for example, RC T-beams depends on many interacting variables. Improvement in predictive ability through ML has far-reaching effects on the design of RC T-beams, particularly regarding estimating shear strength. True prediction of structural properties is a prerequisite for assuring safety and economy in structural design. In addition, designing ML models will cut down the time and computational resources spent on analysis while boosting output reliability^45–48^. Although several studies have applied ML methods to predict the behavior of RC structures, limited research has focused specifically on RC T-beams under complex shear conditions. Moreover, the integration of stochastic optimization techniques such as LF into ML algorithms for structural prediction remains unexplored. To the best of our knowledge, no previous study has combined the DT algorithm with the LF optimization technique to predict shear strength in RC T-beams. While earlier works have applied ensemble methods or traditional regression models, they typically lack mechanisms to overcome local minima or to conduct a global search in the decision space. The novelty of this study lies in introducing a hybrid approach that enhances the generalization capacity of decision trees, making it more suitable for complex nonlinear structural data. By integrating the Levy distribution’s heavy-tailed nature, the proposed method can explore broader solution spaces compared to conventional tree-based models, thereby providing a significant methodological advancement over existing literature.

The present work proposes an investigation into the application of ML techniques in predicting shear strength of RC T-beams. These are Random Forest (RF), Adaptive Boosting (AdaBoost), Decision Tree (DT), K-Nearest Neighbors (KNN), Ridge Regressor, and DT with Levy Flight (Levy-DT). One algorithm, Levy-DT, proposed here as a new algorithm, is the variant of the standard DT algorithm with an enhancement called Levy Flight (LF), a stochastic optimization method that is inspired by natural animal patterns of flight behavior. LF allows the algorithm to explore more broadly by making it easier for the algorithm to escape from local minima. Therefore, it is highly applicable for complex prediction applications such as shear strength estimation^49,50^. The dataset used in this study includes 195 test samples taken from the literature, which gives complete information regarding the properties and shear strength of RC T-beams with different characteristics. The reason ML algorithms are used is to create an extremely accurate prediction model for estimating shear strength in different design situations. The application of the ML technique in this scenario assists in confirming its capability to sculpt design tasks more accurately, reliably, and efficiently. In addition to the creation of models, interpretability for prediction models is achieved through SHAP analysis. SHAP analysis helps explain the influence of each feature, making the model predictions more interpretable^29,43^. This paper utilized SHAP values to show the competence of ML in yielding correct outputs and pinpointing the rudimentary elements that usher in such results. The paper enhances prior informative literature by offering an inquiry into the use of ML techniques in predicting the shear strength of RC T-beams, a significantly vital subject concerning structural engineering, but poorly examined. Unlike traditional methods, this does not require resource-intensive experimental procedures but instead provides a much more efficient and accurate alternative. The present paper contrasts six regression algorithms and pinpoints Levy-DT as the one that excels exceptionally. It further underscores ML model interpretability. SHAP analysis is carried out in the current study to elucidate the relative importance of all input parameters used in predicting shear strength.

## Computational methods and experimental framework

### Dataset description

The statistics for the dataset, which comprises 195 results of RC T-beam tests^51–61^, are presented in Table 1.

**Table 1:** Statistical metrics of experimental data points for RC T beams.

Explanation	Notation	Unit	Minimum	Maximum	Average	Standard deviation
Shear span-effective depth ratio	*a/d*	–	1.46	7.2	3.29	0.89
Web width	*b*	mm	80	457	160.68	56
Flange width	*b*_*f*_	mm	140	1200	508.4	193.8
Flange depth	*h*_*f*_	mm	50	203	91.97	29.37
Effective depth	*d*_1_	mm	140	1092	330.4	129.02
Shear depth	*d*_*v*_	mm	122	989	291.68	125.53
Reinforcement cover depth	*d*_2_	mm	160	1120	348.97	133.43
Beam height	*h*	mm	175	1220	385.61	143.34
Beam length	*L*	mm	860	7520	2798.75	1055.09
Flanged shear span	*a*_*f*_	mm	0	1000	274.68	390.67
Shear span	*a*	mm	265	3350	1082.97	471.74
Loading area width	*l*_*b*1_	mm	30	360	122.42	57.35
Support bearing length	*l*_*b*2_	mm	30	431	104.82	70.49
Longitudinal reinforcement ratio	*ρ*_*s*1_	%	0.49	7.56	3.07	1.41
Longitudinal reinforcement diameter	*ϕ*_*s*1_	mm	10	36	19.3	5.45
Number of longitudinal bars	*n*_*s*1_	-	2	12	5.08	2.1
Yield strength of longitudinal reinforcement	*f*_*y*_	Mpa	340	1860	679.24	452.39
Yield strength of flange reinforcement	*f*_*yf*_	Mpa	262	1860	500	326.73
Flange reinforcement ratio	*ρ*_*s*2_	%	0	2.76	0.77	0.49
Flange reinforcement diameter	*ϕ*_*s*2_	mm	6	29	10.9	3.88
Number of flange reinforcement bars	*n*_*s*2_	-	2	8	4.54	1.64
Maximum aggregate size	*a*_*g*_	mm	10	30	18.3	3.74
Compressive strength of concrete	*f*_*c*_ *‘*	Mpa	12	60	31.12	9.21
Stirrup ratio	*ρ*_*v*_	%	0	1.41	0.22	0.23
Stirrup diameter	*ϕ*_*v*_	mm	3	11	6.39	1.53
Number of stirrups	*n*_*v*_	-	0	3	1.99	0.17
Stirrup spacing	*s*_*v*_	mm	50	500	186.89	79.3
Yield strength of stirrups	*f*_*yv*_	Mpa	223	650	405.19	125.26
Normalized axial stress	*σ = Pu / A*	Mpa	0.45	16.24	2.97	1.93
Ultimate shear force	*V*_*u*_	kN	27	1036	164.75	150.73

The dataset includes 31 independent variables, with the ultimate shear force as the dependent variable. Among these variables, key geometric and mechanical parameters are defined as follows: *a/d* represents the ratio of the shear span to the effective depth. Variable *b* refers to the web width of the beam, whereas variable *bf* denotes the flange width of the beam. The ratio of flange width to web width (*bf*/*b*) and the ratio of flange depth to cross-sectional depth (*h*_*f*_/*h*) are also included as key parameters. Other key measurements include the depth of the flange (*h*_*f*_), effective depth (*d*_*1*_), and shear depth (*d*_*v*_). The distance from the beam to the support corner (*d*_*2*_) and the total length of the beam (*L*) are also noted, along with the distance between flanged shear span (*a*_*f*_) and the distance from the force application point to the beam support (*a*). Additional parameters include the width of the P-force area (*l*_*b1*_) and the distance from the support to the end of the beam (*l*_*b2*_). The reinforcement characteristics include the longitudinal reinforcement ratio (*ρ*_*s1*_), longitudinal reinforcement diameter (⌀_s1_), and number of longitudinal bars (*n*_*s1*_). The details of flange reinforcement are provided by the flange reinforcement ratio (ρ_s2_), diameter (⌀_s2_), Number of flange reinforcement bars (*n*_*s2*_), and yield strength of longitudinal reinforcement (*f*_*y*_). The total gross area of the concrete (*a*_*g*_) and its compressive strength under various conditions (*fc’*) are included as well. Finally, stirrup-related parameters such as the stirrup ratio (*ρ*_*v*_), diameter (*ϕ*_*v*_), number (*n*_*v*_), spacing (*s*_*v*_), and yield strength of stirrups (*f*_*yv*_) are specified, along with the normalized axial stress (*σ*) and ultimate shear force (*V*_*u*_) of the beam. *σ* refers to the axial load (*P*_*u*_) divided by the gross cross-sectional area of the beam, representing the intensity of axial load relative to the section size.

**Fig. 1:**
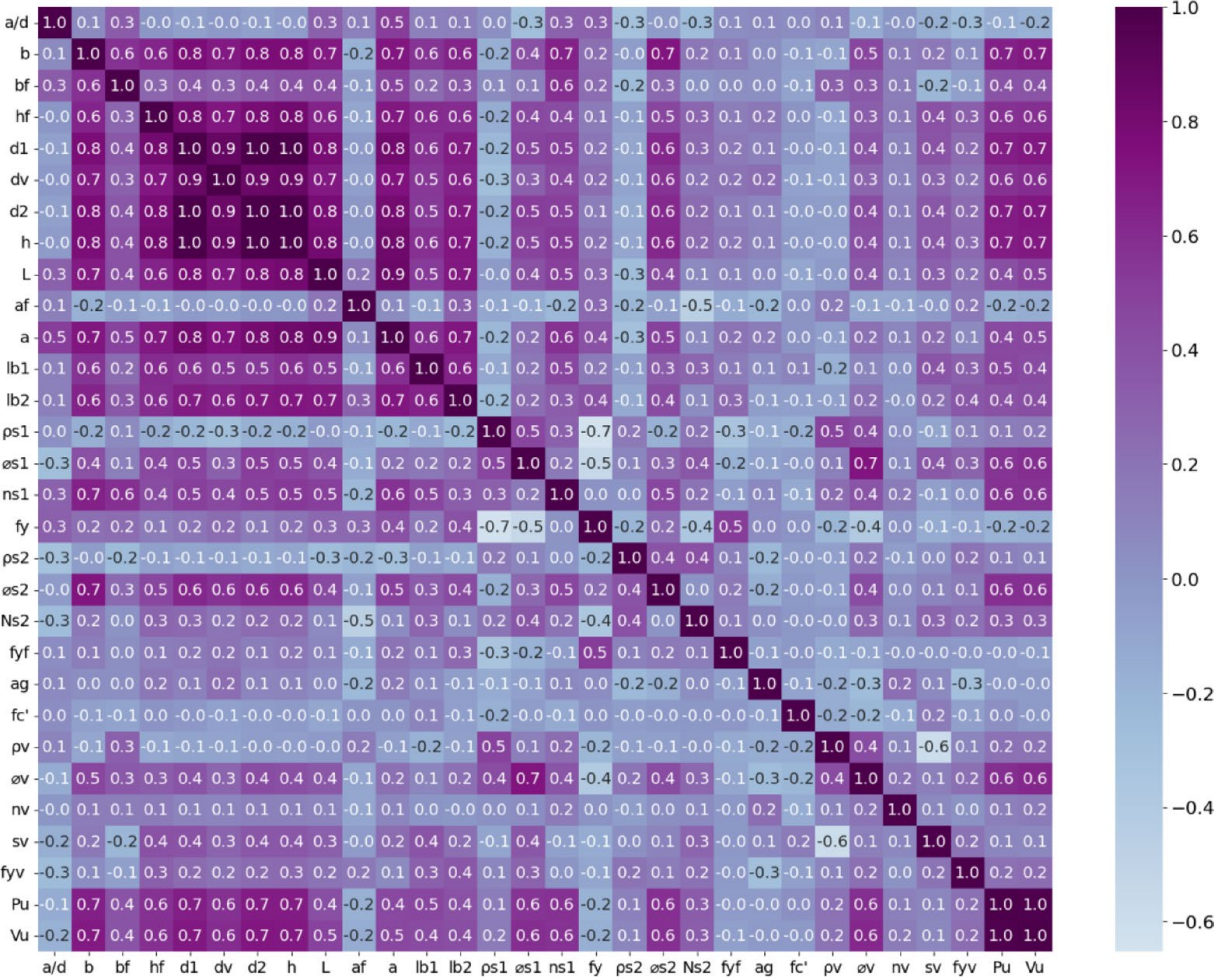
Correlation matrix of variables in the dataset.

The dataset used in this study is initially explored using a correlation matrix, as illustrated in Fig. 1. This chart is a key tool that assesses the degree of linear relationships among variables, assisting in data preprocessing. Correlation values range from + 1 (indicating perfect positive correlation) to -1 (perfect negative correlation), with 0 implying no linear association. When analyzed in relation to the target variable ultimate shear force the matrix highlights several key variables that exhibit significant correlation, providing insight into the underlying factors influencing shear strength. The bottom row of the matrix provides a comprehensive view of these correlations. The most substantial positive correlations with Vu are observed for beam width (*b*), beam height (*h*), effective depth (*d*_1_), reinforcement cover depth (d_2_), and shear depth (*d*_*v*_), all of which show correlation coefficients between 0.6 and 0.7. This strong relationship confirms that the geometric dimensions of the cross-section play a dominant role in determining shear capacity, which aligns with fundamental structural engineering principles where larger cross-sections typically provide greater resistance to shear forces. The longitudinal reinforcement characteristics show moderate positive correlations with Vu: the reinforcement diameter (*ø*_*s*1_) has a correlation coefficient of 0.6, while the number of longitudinal bars (*n*_*s*1_) shows a coefficient of approximately 0.6. This suggests that longitudinal reinforcement contributes to shear resistance through dowel action and improved crack control. For transverse reinforcement parameters, stirrup diameter (*ø*_*v*_) correlates more strongly with *V*_*u*_ (0.6) compared to the stirrup ratio (*ρ*_*v*_) which shows a weaker positive correlation (0.2). This might indicate that in the dataset, the actual diameter of stirrups has more consistent influence on shear capacity than the volumetric ratio alone. The shear span-to-depth ratio (*a/d*) exhibits a negative correlation with *V*_*u*_ (-0.2), confirming the well-established principle that beams with shorter shear spans relative to their depth demonstrate higher shear resistance due to the development of arch action. The correlation between flange width (*b*_*f*_) and *V*_*u*_ (0.4) indicates that the T-beam configuration provides a moderate enhancement to shear capacity, likely through the contribution of the flange to the compression zone and modified stress distribution. This systematic examination of correlations between various parameters and *V*_*u*_ provides valuable insights for feature selection in predictive modeling and reinforces the complex, multifaceted nature of shear behavior in RC T-beams.

### Machine learning architecture and implementation

This research utilizes six independent ML techniques DT, RF, KNN, Ridge Regression, AdaBoost, and the proposed Levy-DT to predict the shear strength of RC T-beams. All the techniques are used due to their power in modeling complex relationships. The selection of these specific ML algorithms is driven by their diverse learning paradigms and proven effectiveness in regression tasks^62^. DT and RF are chosen for their capability to handle nonlinear relationships and their interpretability, which is essential for understanding the influence of input features on shear strength. The DT technique is notably used in classification and regression though it caters to a vast range of data predicaments^63,64^. It constructs tree-like models of decisions by partitioning the data according to the characteristics defined in the model. Features are indicated at internal nodes; edges show the associated decisions; leaf nodes represent outcomes. Recursive partitioning of the dataset into subsets of samples according to some splitting criterion until an achieved termination point is found contributes to the construction of the tree. Either the depth of the tree or the minimum number of samples allowed at each node can be considered as part of the termination criterion. RF is one such advanced analytical method that has come into being through years of statistical exploration and computational progress^65,66^. Each computational tree within this forest grows independently, drawing unique data samples and evaluating disparate feature combinations. This architectural design is not merely a technical trick,it is a profound strategy for mitigating predictive uncertainties. KNN offers a non-parametric approach that captures local data structures, making it a valuable benchmark. It is a non-parametric method widely used for regression and classification tasks. It estimates the target value of a sample based on the average of the *k* most similar training instances, where similarity is typically measured using Euclidean distance^67–70^. The performance of KNN depends heavily on the selection of the parameter *k*: small values may lead to high variance, while large values may smooth out important local patterns. Ridge Regression is included as a regularized linear model to evaluate the performance of simpler, yet robust, predictive strategies. It introduces a penalty term to the loss function proportional to the square of the coefficients, thereby shrinking them and reducing variance. This results in more stable and generalizable models^71,72^. AdaBoost is utilized to assess the contribution of ensemble learning through boosting, which enhances predictive accuracy by focusing on difficult-to-predict instances. It is an ensemble learning technique that unites all weak learners into one strong learner thus enhancing performance^73^. It involves training several models using a sequential approach where each model targets the errors of the previous ones. Weights are assigned to misclassified instances and future models are created with such difficult cases in mind. AdaBoost updates the weights of the data points based on how well they perform in the prior model, resulting in a weighted sum of all predictions.

**Fig. 2:**
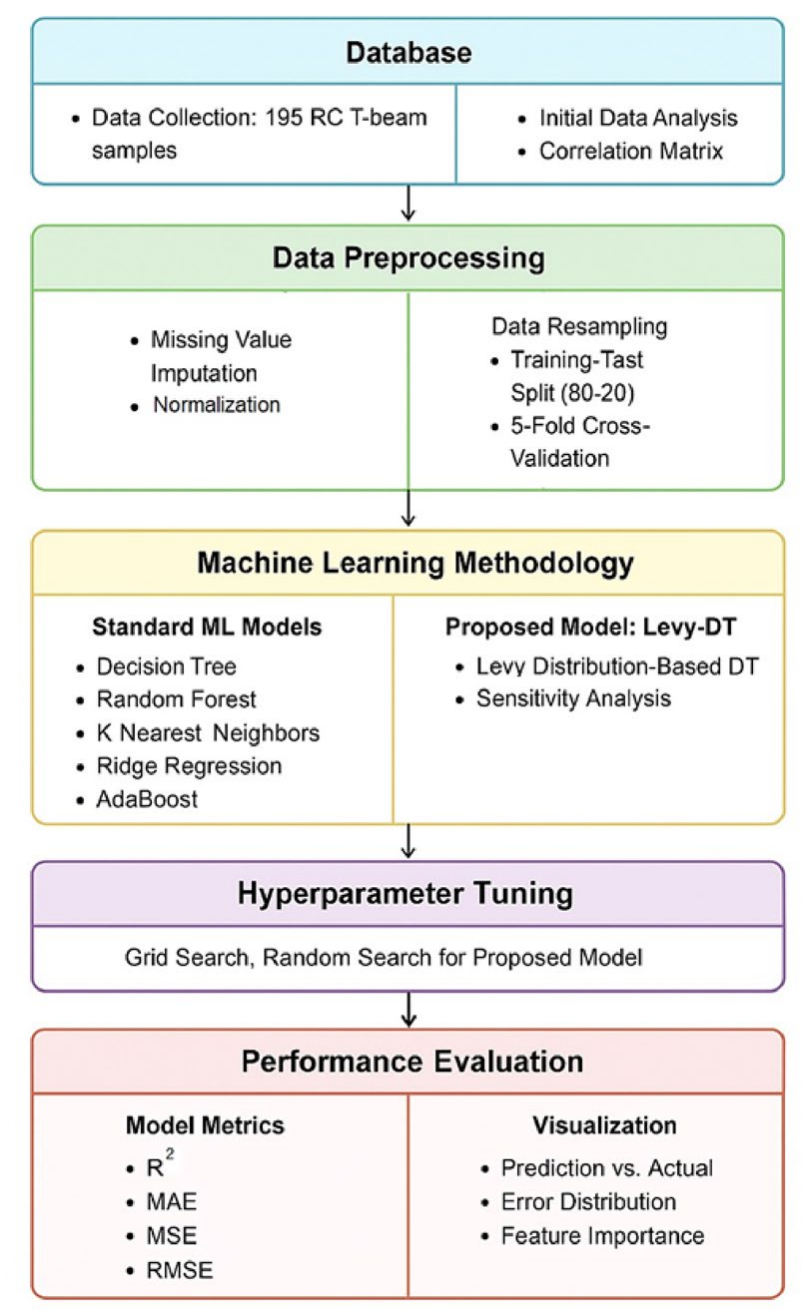
Methodological framework of the Levy-DT-based predictive model.

A significant contribution of this study is the introduction of Levy-DT, an advanced extension of traditional DT that effectively addresses the limitations of standard DT architectures. It integrates the exploratory strength of LF with tree-based learning, aiming to improve prediction accuracy and generalization by balancing exploration and exploitation during model optimization. Specifically, Levy-DT mitigates the prevalent issue of DT models becoming trapped in local minima when processing complex datasets, thereby enhancing overall performance and reliability. Figure 2 presents the overall workflow of the proposed predictive modeling framework based on the Levy-DT algorithm. The diagram outlines a comprehensive and systematic sequence of steps used to predict the shear strength of RC T-beams using both standard and advanced ML techniques. The methodology commences with data compilation, encompassing the gathering of 195 RC T-beam specimens alongside preliminary statistical examination, including correlation matrix analysis to identify interdependencies between input parameters. Subsequently, during the data preparation stage, absent values undergo imputation while feature standardization is performed to achieve consistent scaling across variables. The compiled dataset undergoes partitioning into training and testing portions using an 80 − 20 distribution, with 5-fold cross-validation implemented to validate model reliability and ensure adequate generalization capabilities. The ML methodology is split into two parallel streams: one employing standard ML models (e.g., DT, RF, KNN, Ridge Regression, AdaBoost) and the other focusing on the proposed Levy-DT model, which integrates a Levy distribution-based stochastic optimization method to enhance the standard DT algorithm. To refine model performance, the proposed methodology incorporates sensitivity analysis and systematic hyperparameter tuning. Both grid search and random search techniques are employed to identify optimal parameter configurations for the Levy-DT model, balancing accuracy and computational efficiency. Model performance is then evaluated using widely accepted regression metrics, including R², MAE, MSE, and RMSE. In addition, several visual diagnostics such as prediction vs. actual plots, error histograms, and feature importance rankings are used to evaluate model fidelity and explainability. These tools collectively validate the effectiveness of the proposed approach relative to other ML algorithms.

Table 2 outlines the key structural and hyperparameter settings for each ML model tested in the study. This includes conventional algorithms from the scikit-learn library as well as the proposed Levy-enhanced decision tree (Levy-DT). Where applicable, key training parameters such as the number of estimators, depth, regularization strength, and neighborhood size are explicitly defined. In particular, the Levy-DT model is a custom-developed regression algorithm that extends the DecisionTreeRegressor from scikit-learn by integrating an LF-based optimization mechanism. All relevant parameters for the Levy-DT model including Levy_lambda (*λ*), step size (*β*), num_iterations, max_depth (*D*_*max*_), and random_state are also provided in Table 2.

**Table 2:** Topological structure and hyperparameters of the ML models.

Model name	Library/implementation	Key parameters/topology
DT	sklearn.tree.DecisionTreeRegressor	criterion=’squared_error’, splitter=’best’, max_depth = None, random_state = 10
RF	sklearn.ensemble.RandomForestRegressor	n_estimators = 10, criterion=’squared_error’, max_features=’sqrt’, random_state = 10
AdaBoost	sklearn.ensemble.AdaBoostRegressor	n_estimators = 50, learning_rate = 1.0, random_state = 10
KNN	sklearn.neighbors.KNeighborsRegressor	n_neighbors = 5, weights=’uniform’, metric=’minkowski’, *p* = 2
Ridge	sklearn.linear_model.Ridge	alpha = 0.01, solver=’auto’, random_state = 10
Levy-DT	Custom class based on sklearn.tree.DecisionTreeRegressor with LF-based optimization	λ = 1.3, step size (β) = 0.04, num_iterations = 15, D_max_ =10, random_state = 10

### Proposed Levy flight-enhanced decision tree algorithm

The Levy Flight-Enhanced Decision Tree (Levy-DT) algorithm signifies an enhancement over traditional DT algorithms by integrating the LF optimization method. Conventional DT models generally utilize feature and threshold selection techniques that minimize loss functions, frequently resulting in locally optimum solutions susceptible to overfitting and deficient in global exploration. The LF method mitigates this constraint by using a stochastic process defined by random, long-range leaps. These transitions enable a more comprehensive search of the solution space, improving the algorithm’s capacity to surpass local optima and detect globally optimal splits in regression problems. The fundamental principle of LF is rooted in its unique probability distribution^74,75^. LF is a type of random walk in which the step lengths $\:L$ follow a heavy-tailed distribution, typically a power-law:

1 $$L \sim \frac{1}{{\left| s \right|^{{1 + \beta }} }}~~with1 < \beta \le 3,$$

where $\:s$ represents the step size, and $\:\beta\:$ is the Levy index, controlling the jump frequency and step size distribution. Unlike conventional DTs that finalize splits based on immediate impurity reduction, Levy-DT treats the initial greedy solution as a starting point for further stochastic exploration, fundamentally changing the threshold selection paradigm. Rather than incrementally adjusting splits, it allows threshold candidates to “jump” to distant values within the feature domain, enabling the algorithm to escape local minima and construct more optimal tree structures. In practical terms, the integration of LF into the DT framework is achieved through a two-phase threshold optimization process at each decision node. This process directly addresses the question of how LF enhances traditional DT splitting: the algorithm does not replace conventional greedy search but rather perturbs the optimal split points found by traditional methods to explore additional candidate thresholds. First, the standard DT mechanism selects a set of initial candidate split points* T*_0_={*t*_1_,* t*2, …,* t*_k_} based on impurity measures such as mean squared error (MSE). Then, instead of finalizing the best split solely from this set, the algorithm applies Levy-based stochastic perturbations to these thresholds. For each initial threshold *ti*, a new set of candidate splits is generated using the following formulation:

2 $$\:{t}_{i,new}={t}_{i}+\alpha\:\cdot\:Levy\left(\lambda\:\right)\cdot\:({t}_{max}-{t}_{min})$$

Here, *α* is a scaling factor, Levy(*λ*) is a sample from a Levy distribution with stability parameter *λ*, and the feature range (*t*_*max*_*−t*_*min*_) serves to normalize the step magnitude. The interaction between these parameters is crucial for algorithm performance: *α* controls the magnitude of perturbations, *λ* determines the probability of large versus small jumps through the Levy stability parameter, while the feature range normalization $\:({t}_{max}-{t}_{min})$ ensures that perturbations are proportional to the actual feature scale, maintaining algorithmic consistency across different feature domains. This formulation allows the algorithm to perform both local refinements and global explorations of the threshold space, increasing the likelihood of locating globally optimal splits.

All thresholds both original and perturbed are evaluated using an expanded cost function that balances prediction error and model complexity:

3 $$C = \mathop \sum \limits_{{i = 1}}^{n} \left( {y_{i} - \hat{y}_{i} } \right)^{2} + \gamma \left| s \right|,$$

where $\:{y}_{i}$​ and $\:{\widehat{y}}_{i}$ are the observed and predicted values, respectively, $\:n$ is the number of data points, and *γ* represents a regularization parameter that facilitates control over the complexity of the tree. The term *s​* represents the LF step size applied to the current node’s threshold, calculated as *s = α · Levy*(*λ*) *·* (*t*_*max*_ *- t*_*min*_). The core of the study introduces a two-stage optimization process within the Levy-DT framework. At each decision node, a stochastic perturbation term *s*, governed by a heavy-tailed Levy distribution, is applied. For small* s* values, the model makes fine-grained adjustments; larger values facilitate broader shifts in decision thresholds. This mechanism enables the algorithm to explore both local and global areas of the solution space, mitigating the risk of local optima a frequent issue with conventional decision tree models. To prevent excessive deviation, a regularization factor* γ*/*s*/ is incorporated, maintaining interpretability while preserving adaptability. Since* s* is sampled from a distribution characterized in Eq. (1), the approach seamlessly blends exploratory and exploitative strategies in the decision-making process, enabling more effective partitioning of nonlinear and high-dimensional feature spaces than traditional trees can achieve. As a result, it addresses a well-known limitation in tree-based algorithms, namely their vulnerability to local optima that compromise generalization in nonlinear prediction tasks.

In contrast to prior studies that utilize fixed impurity-based splits or greedy heuristics, the Levy-DT approach dynamically searches the split thresholds across the global solution space, leading to superior regression tree structures. This global search behavior, guided by Levy-distributed jumps, allows the model to escape local optima an issue that conventional DTs or boosted trees often encounter. Therefore, the core innovation of Levy-DT lies in embedding stochastic, biologically inspired search processes within deterministic ML structures, which is a novel contribution in the field of structural engineering applications.

## Comparative analysis of ML algorithms for shear strength prediction

This section presents a comparative analysis of six ML algorithms utilized to predict the shear strength of RC T-beams. The algorithms include AdaBoost, DT, RF, KNN, Ridge, and the novel algorithm, Levy-DT, which enhances the standard DT using the LF mechanism. The proposed Levy-DT aims to improve the accuracy of prediction, offering a more robust approach for complex structural design scenarios. The input parameters include several geometrical and material properties of the T-beams, such as shear span-depth ratio (*a/d*), web width (*b*), flange width (*bf*), flange depth (*hf*), reinforcement ratios (*ρ*_*s*1_, *ρ*_*s*2_), and concrete compressive strength (*fc*’). The output is the ultimate shear force (*Vu*), which is related to the shear strength of the beam and represents the internal force acting to resist sliding along the cross-section of the beam. Data preprocessing is performed in several stages to ensure reliable model training and testing. First, missing data is addressed using “*SimpleImputer*” with a mean strategy, which replaces gaps in the dataset with the mean value of each respective feature^76^. This imputation approach is adopted due to the relatively low proportion of missing data and the approximately symmetric distribution of most features. It preserved the statistical characteristics of the dataset while preventing bias introduced by deletion or arbitrary imputation techniques. Next, feature scaling is implemented using “*StandardScaler*” to normalize all input variables to a common scale with zero mean and unit variance, which is particularly important for distance-based algorithms and improves convergence speed for most ML models. The experimental design incorporated both traditional train-test splitting and “*k-fold cross-validation*” to ensure model robustness. Initially, 80% of the dataset (156 samples) is designated for model training, while the remaining 20% (39 samples) is reserved for testing. Additionally, a “5-fold cross-validation” approach is implemented to evaluate model performance stability across different data subsets, with each fold maintaining the same preprocessing pipeline to prevent data leakage. All computations and model development, including implementation of the Levy-DT algorithm, are conducted using Python^77^.

To ensure a fair and rigorous comparison between the proposed Levy-DT and baseline models, hyperparameter optimization is implemented for the standard DT algorithm. This optimization process aims to identify the most suitable regularization parameters that balance model complexity with generalization capability. The hyperparameter optimization is conducted using GridSearchCV with 5-fold cross-validation to systematically evaluate different parameter combinations. The optimization space includes critical regularization parameters: max_depth values ranging from 5 to 15 plus unrestricted depth, min_samples_split values from 2 to 15, min_samples_leaf values from 1 to 8, max_features options including square root, logarithmic, and all features, and min_impurity_decrease thresholds from 0.0 to 0.002. A balanced model selection approach is employed to address potential overfitting concerns. Rather than selecting the model with the highest cross-validation score alone, the selection criterion incorporated both test performance and the overfitting gap between training and test scores. To ensure a reasonable level of generalization alongside competitive predictive accuracy, the model selection strategy incorporates both training and validation performance. Initially, the five most promising models identified through cross-validation results are shortlisted for further evaluation. For each of these candidates, R² scores are computed on both the training and test sets to quantify potential overfitting. The performance gap, expressed as the difference between these two scores, serves as a measure of model robustness. A balance score is then calculated by adjusting the test R² value with a penalty proportional to the overfitting gap, particularly when the gap exceeds a threshold of 0.1. The model achieving the highest balance score is ultimately selected, reflecting an optimal compromise between accuracy and generalization. This systematic optimization process provides a robust baseline for comparison with the proposed Levy-enhanced approach, allowing for meaningful evaluation of the enhancement achieved through LF integration.

**Fig. 3:**
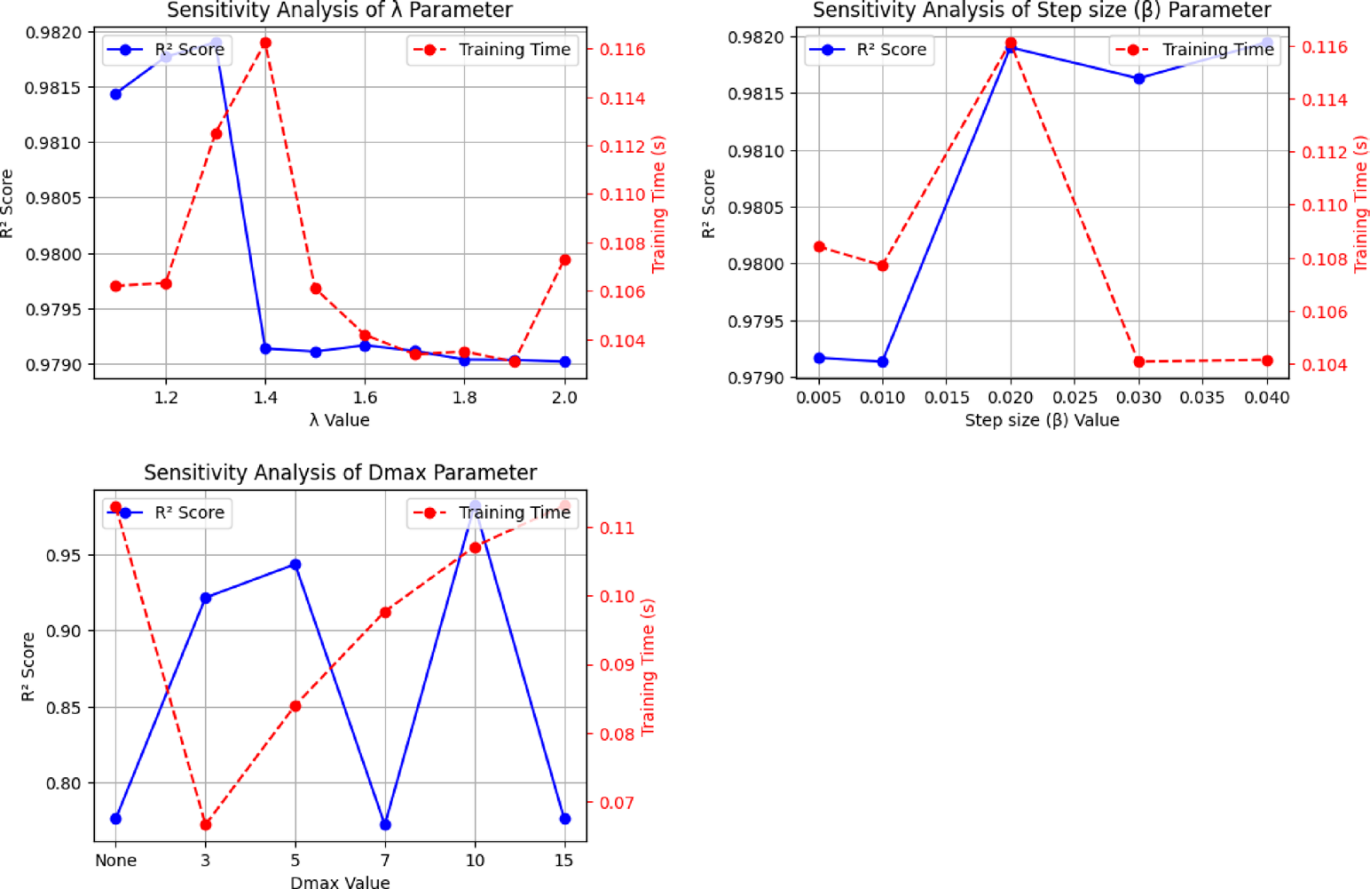
Sensitivity analysis of λ, β, and Dmax parameters for the Levy-DT algorithm.

The sensitivity analysis conducted on the Levy-DT algorithm, as visualized in Fig. 3, provides valuable insights into the influence of key parameters on model performance and computational efficiency. This analysis addresses the relationship between parameter selection and model performance metrics, particularly the R² score and training time.

The analysis of the *Levy lambda (λ)* parameter reveals a complex relationship with model performance. As illustrated in the first graph, the R² score demonstrates notable sensitivity to *λ* variations, with peak performance occurring at *λ* = 1.3 (R² ≈ 0.979). Lower *λ* values (1.1–1.3) generally yielded superior predictive performance compared to higher values (1.4-2.0), where performance stabilized at a lower level (R² ≈ 0.979). This indicates that the stochastic search behavior governed by *λ* exhibits an optimal range for this specific dataset. Training time exhibits an irregular pattern across *λ* values; notably, at *λ* = 1.3 where the highest R² score is observed the training time remains at a moderate level, indicating a favorable balance between performance and computational cost.

The *β* parameter demonstrates a pronounced effect on model performance, with optimal R² scores observed at a *β* value of 0.02. However, the difference in R² between *β* = 0.02 and *β* = 0.04 is minimal (Δ ≈ 0.0001). Considering that *β* = 0.04 yields the shortest training time, it is ultimately selected to balance predictive performance and computational efficiency. This choice reflects a deliberate trade-off between near-optimal accuracy and significantly improved training speed. The performance trend shows an initial improvement as *β* increases from 0.005 to 0.02, followed by a slight decrease at higher values, suggesting that intermediate perturbation magnitudes enable effective exploration of promising solution regions without excessive deviation from original feature values.

Among all the parameters investigated, the maximum tree depth (*D*_*max*_) demonstrated the most significant effect on predictive outcomes. R² values showed considerable fluctuation across different depth limits, with notable improvements observed at depths of 5 and 10, and declines at depth 7 and when no upper limit is applied. This non-monotonic relationship underscores the complexity of tree-based model capacity optimization and the importance of careful depth selection to prevent both underfitting and overfitting. Training time generally increases with greater *D*_*max*_ values, reflecting the expected computational cost of building deeper trees. While a general upward trend is visible, the pattern is not strictly linear. In fact, some configurations such as *D*_*max*_ = 10 achieve high R² values with only a moderate increase in training time, indicating that optimal depth values can maintain computational efficiency when well aligned with the LF-guided search.

Based on comprehensive sensitivity analysis, the optimal parameter configuration is determined to be *λ* = 1.3, *β* = 0.04, *D*_*max*_ = 10, with 15 iterations. This configuration achieved an R² score of 0.979, RMSE of 26.962, and MAE of 14.446, with a training time of 0.1193 s. When compared to standard DT methods, the Levy-DT algorithm introduces additional computational overhead due to the stochastic perturbation process and multiple iterations. However, the sensitivity analysis demonstrates that this overhead can be minimized through judicious parameter selection while still achieving superior predictive performance. The modest training time of 0.1193 s for the optimal configuration suggests that the LF enhancement mechanism introduces acceptable computational costs relative to the performance benefits gained. The parameter tuning process reveals the delicate balance between model performance and computational efficiency in the Levy-DT algorithm. The optimal configuration achieves superior predictive accuracy compared to standard DT while maintaining reasonable computational demands, validating the practical applicability of the LF enhancement mechanism in tree-based regression tasks.

**Fig. 4:**
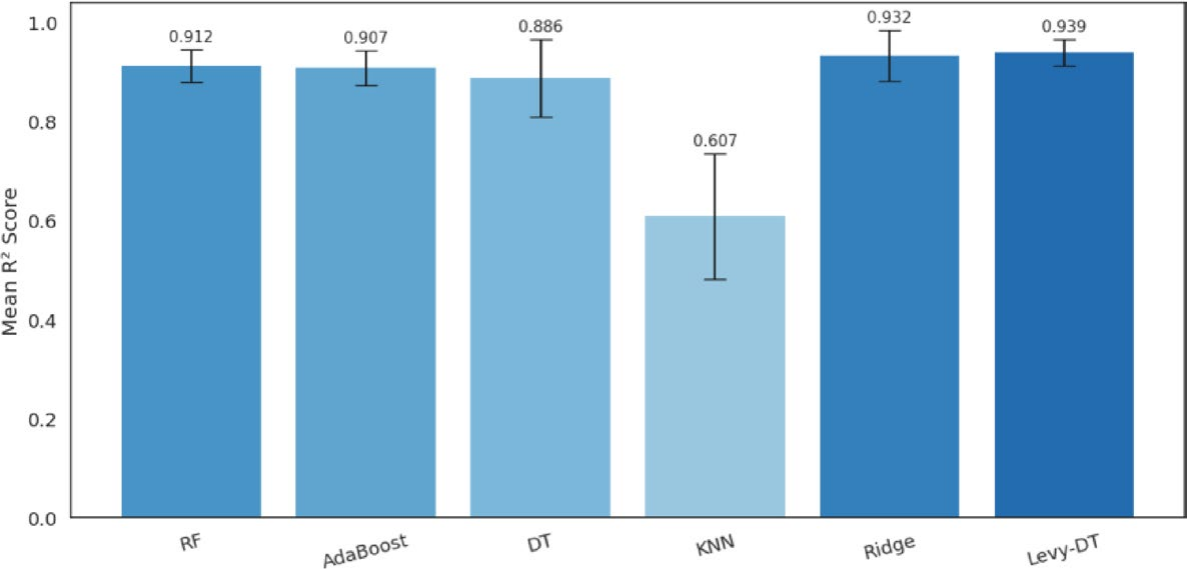
Model performance evaluation with 5-Fold Cross-validation.

The cross-validation analysis implemented in the study, the results of which are presented in Fig. 4, represents a robust methodology for evaluating model performance across different data subsets. The k-fold cross-validation procedure is systematically implemented with several key characteristics. The dataset is partitioned into five equal subsets (folds), with each fold serving as a validation set once while the remaining folds form the training set. Within each fold iteration, crucial preprocessing steps are applied independently to prevent data leakage. Missing values are imputed using the mean strategy via SimpleImputer, and features are standardized using StandardScaler to ensure zero mean and unit variance. For each fold, models are trained on the preprocessed training subset with consistent hyperparameters. Standard models (RF, AdaBoost, KNN, Ridge) are trained with their default parameters, while both DT and Levy-DT models utilize optimized hyperparameters determined through systematic parameter tuning, with DT employing regularization parameters identified via GridSearchCV and Levy-DT utilizing optimized parameters (λ, scale, maximum depth) determined from the sensitivity analysis. The R² score is calculated for each fold’s validation set, capturing the model’s ability to explain variance in unseen data. Figure 4 shows that the proposed Levy-DT algorithm achieved the highest mean R² score (0.939) across all folds, demonstrating its robust predictive capability for shear strength estimation in RC T-beams. The performance hierarchy is clearly established with Levy-DT (0.939) and Ridge (0.932) displaying superior performance, RF (0.912) and AdaBoost (0.907) showing good but slightly reduced predictive power, DT (0.886) providing moderate performance, and KNN (0.607) significantly underperforming compared to other algorithms. The error bars represent standard deviation across folds and indicate that Levy-DT and Ridge demonstrate high stability with small error bars, KNN shows considerable variability suggesting sensitivity to specific data partitions, while other algorithms maintain relatively consistent performance across different data subsets. The cross-validation results substantiate the effectiveness of the LF enhancement to the DT algorithm. The marginal improvement of 6.2% in R² score over the standard DT (from 0.877 to 0.939) indicates that the stochastic perturbation mechanism successfully mitigates local optima issues in the base algorithm. Furthermore, the consistency across folds confirms that the performance improvement is not coincidental but rather a systematic enhancement provided by the LF mechanism.

Upon examination of the test data, as presented in Table 3, it is evident that the Levy-DT algorithm exhibits superior performance among the evaluated models. It achieves the highest R² value of 0.982, indicating strong predictive accuracy. Furthermore, the model yields the lowest RMSE (27.941) and MSE (780.698) values, reflecting its excellent capability to minimize prediction errors. The MAE of 14.551 further confirms its precision in estimating the shear strength of RC T-beams. The optimized DT model, which underwent systematic hyperparameter tuning to ensure fair comparison, demonstrates improved generalization capability compared to an unconstrained baseline. However, even with optimization, the DT model achieves a moderate R² of 0.731 with corresponding RMSE (97.281) and MSE (9463.552) values that are substantially higher than those of Levy-DT. This performance gap highlights the effectiveness of the LF enhancement in further improving the DT algorithm’s predictive capability beyond conventional optimization approaches. The Ridge regression model demonstrates solid performance with an R² of 0.906, RMSE of 57.544, and MSE of 3311.308. While not as accurate as Levy-DT, it performs better than ensemble methods such as RF (R² = 0.847) and AdaBoost (R² = 0.827), which show higher error values. KNN, on the other hand, yields the weakest test results with an R² of 0.730, RMSE of 97.490, and MSE of 9504.266, indicating that it struggles to capture the nonlinearities of the dataset.

**Table 3:** Test performance evaluation of regression models.

Algorithm	*R* ^2^	MSE	RMSE	MAE
Levy-DT	**0.982**	**780.698**	**27.941**	**14.551**
DT	0.731	9463.552	97.281	31.515
Ridge	0.906	3311.308	57.544	27.740
RF	0.847	5379.970	73.348	23.831
AdaBoost	0.827	6091.706	78.049	34.920
KNN	0.730	9504.266	97.490	43.954

Significant values are given in bold.

As detailed in Table 4, performance on the training dataset reveals important insights about model behavior and generalization capability. The optimized DT model, despite systematic hyperparameter tuning aimed at preventing overfitting, still achieves very high training performance (R² = 0.986, RMSE = 9.988, MSE = 99.753, MAE = 5.250). However, when compared to its test performance (R² = 0.731, RMSE = 97.281 in Table 3), a substantial performance gap remains evident, indicating that even with regularization, complete elimination of overfitting remains challenging for the standard DT algorithm. In contrast, the Levy-DT model demonstrates consistently high performance across both training and test datasets with minimal performance degradation, suggesting that the incorporation of LF-based perturbations provides an additional layer of regularization that effectively enhances generalization beyond conventional hyperparameter optimization approaches. This consistent performance across training and testing phases validates the effectiveness of the stochastic perturbation mechanism in mitigating overfitting tendencies inherent in tree-based algorithms. The Ridge model performs exceptionally well during training (R² = 0.990, RMSE = 13.860), demonstrating its inherent stability and regularization effectiveness. Similarly, RF (R² = 0.982) and AdaBoost (R² = 0.969) exhibit strong training accuracy with acceptable error metrics, benefiting from their ensemble nature that naturally provides regularization through model averaging and boosting mechanisms respectively. KNN, by contrast, shows considerably higher RMSE (64.768) and MSE (4194.849) during training, along with a relatively lower R² of 0.784, indicating a weaker fit even on the training data. This poor performance can be attributed to several factors. First, the feature space characteristics of the dataset make distance-based algorithms such as KNN less effective, as the Euclidean distance metric fails to capture the complex relationships between features and target variables in this context. Moreover, the dimensionality of the dataset poses challenges for KNN, as the algorithm suffers from the “curse of dimensionality” where distance measurements become less meaningful in higher-dimensional spaces. The comparison with other algorithms, particularly tree-based methods such as DT and Levy-DT, demonstrates the limitations of purely distance-based approaches for this specific prediction task, where hierarchical decision boundaries prove more effective than neighborhood-based predictions.

**Table 4:** Train performance evaluation of regression models.

Algorithm	*R* ^2^	MSE	RMSE	MAE
Levy-DT	**0.995**	**99.753**	**9.988**	**5.250**
DT	0.986	263.12	16.221	7.695
Ridge	0.990	192.095	13.860	10.124
RF	0.982	340.426	18.451	7.694
AdaBoost	0.969	594.192	24.376	21.354
KNN	0.784	4194.849	64.768	32.610

Significant values are given in bold.

**Fig. 5:**
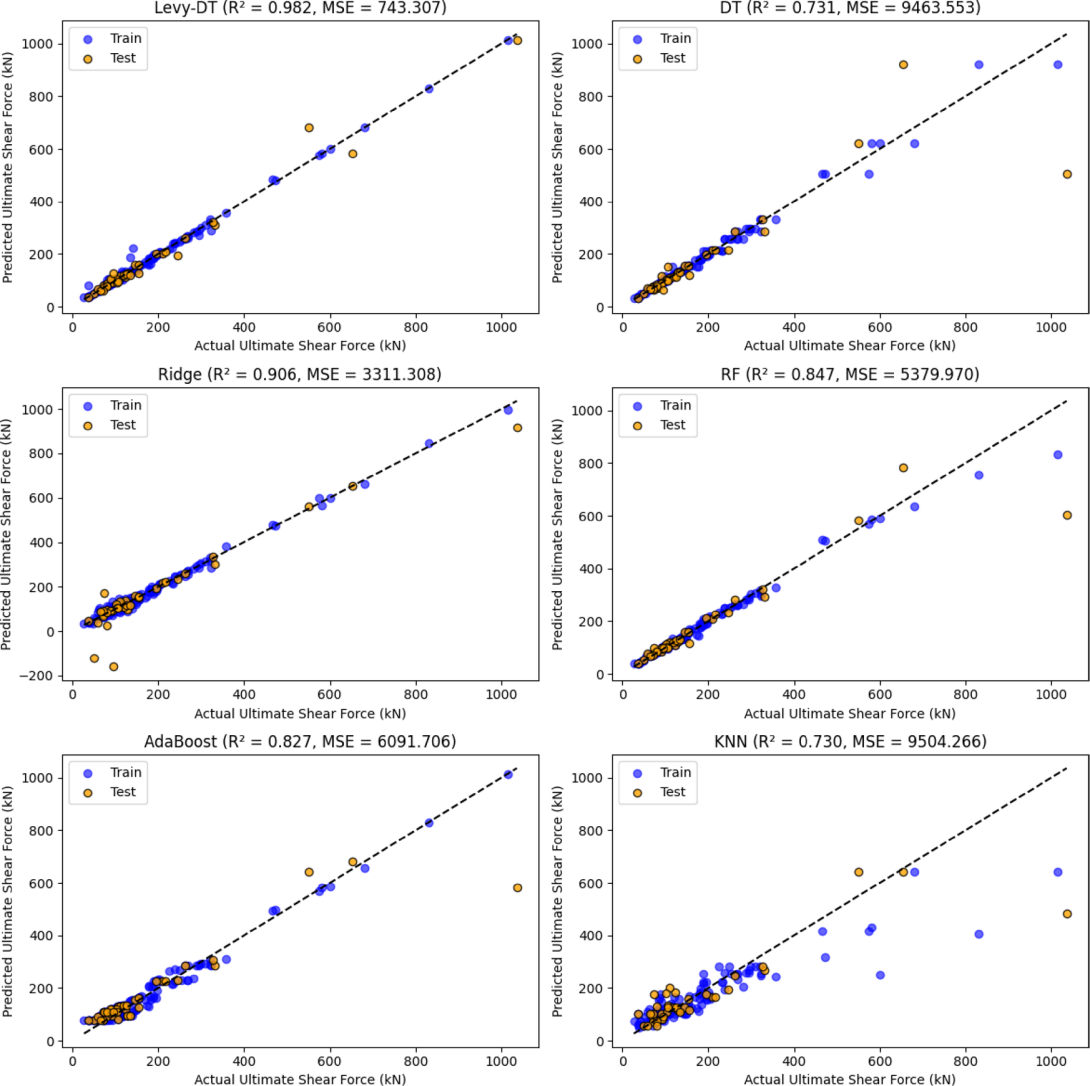
Performance evaluation of different regression algorithms in shear strength prediction.

Figure 5 compares the predicted values with the true values for both the training and test datasets across the algorithms employed. Each subplot visualizes the alignment of the predicted values (y-axis) with the true values (x-axis), where the dotted diagonal line represents perfect predictions. The Levy-DT algorithm demonstrates a strong agreement between the predicted and true values for both the training (green) and test (blue) datasets, as most points lie close to the diagonal line. This reflects the superior performance of the algorithm in accurately capturing underlying data patterns with excellent generalization capability. The optimized DT algorithm exhibits reasonable correspondence between the predicted and true values in the training dataset; however, it presents a noticeable increase in deviation in the test dataset, indicating a performance gap between training and testing phases despite hyperparameter optimization. This performance difference highlights the inherent limitations of conventional regularization techniques in completely addressing generalization challenges in DT algorithms, thereby demonstrating the added value of the LF enhancement mechanism. The Ridge and RF algorithms exhibit a moderate degree of alignment, with discernible deviations from the diagonal line, especially within the test set, which indicates a comparatively lower level of predictive accuracy. AdaBoost also performs reasonably well, though its predictions for the test data deviate more from the true values compared to the tree-based algorithms, signaling its relatively lower accuracy for this specific dataset. The KNN algorithm has the most scattered predictions, particularly for larger values, highlighting its weaker performance compared to the other models. This is consistent with the lower R² and higher error metrics observed for KNN in the test data, as it struggles to generalize well to the complex relationships in the dataset. In general, the figure visually reinforces the quantitative results from the tables, emphasizing the robustness of Levy-DT in providing accurate predictions with superior generalization capability, particularly in comparison to both the optimized baseline DT and other comparative methods.

**Fig. 6:**
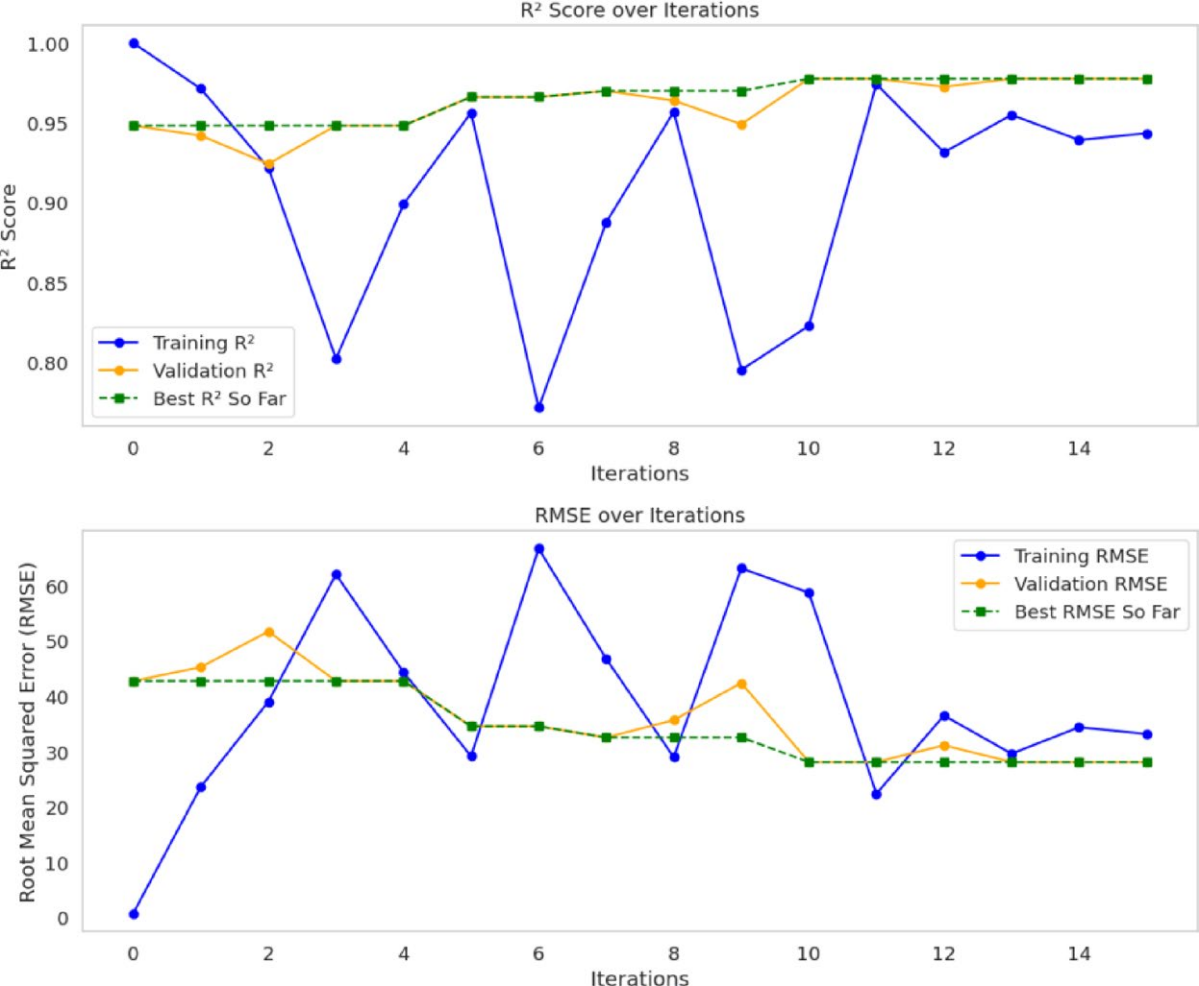
Convergence behavior of the Levy-DT model over training iterations.

Figure 6 presents the performance of the Levy-DT model across 15 iterations using two key metrics: the R² score and the RMSE. Initially, the R² score plot reveals a perfect score (1.0) for the training data at iteration 0, corresponding to the standard DT model, while the validation score remains lower, around 0.95. This discrepancy indicates the standard DT’s tendency to overfit the training data. However, as the iterations progress, the “Best R² So Far” curve shows a gradual increase, approaching 0.98. This curve represents the best performance obtained up to each iteration and illustrates how the Levy-DT algorithm progressively improves its generalization capability. Notably, substantial fluctuations are observed between the training and validation R² scores at several iterations (e.g., the 3rd, 5th, and 9th). These variations are attributed to the stochastic nature of LF-based perturbations. Nonetheless, the applied improvement strategies enable the algorithm to recover from such instabilities, and the “Best R² So Far” curve maintains consistently high performance. A similar trend is evident in the RMSE plot. Initially, the training RMSE is very low (close to 0), whereas the validation RMSE is significantly higher. As the iterations proceed, the gap between the training and validation RMSE tends to narrow, indicating an enhancement in the model’s generalization performance. Similar to the R² score plot, the RMSE values also exhibit fluctuations at certain iterations, consistent with the inherent randomness introduced by the Levy mechanism. After the 10th iteration, both training and validation metrics become more stable, suggesting that the Levy-DT algorithm begins to converge and achieves optimal performance. At this stage, the best R² score stabilizes around 0.98, while the difference between training and validation metrics is minimized. This convergence analysis highlights a significant advantage of the Levy-DT model: it effectively mitigates the overfitting issue commonly observed in standard DTs, while consistently enhancing generalization across iterations. These findings demonstrate that Levy-DT can offer reliable and stable performance in complex regression tasks.

**Fig. 7:**
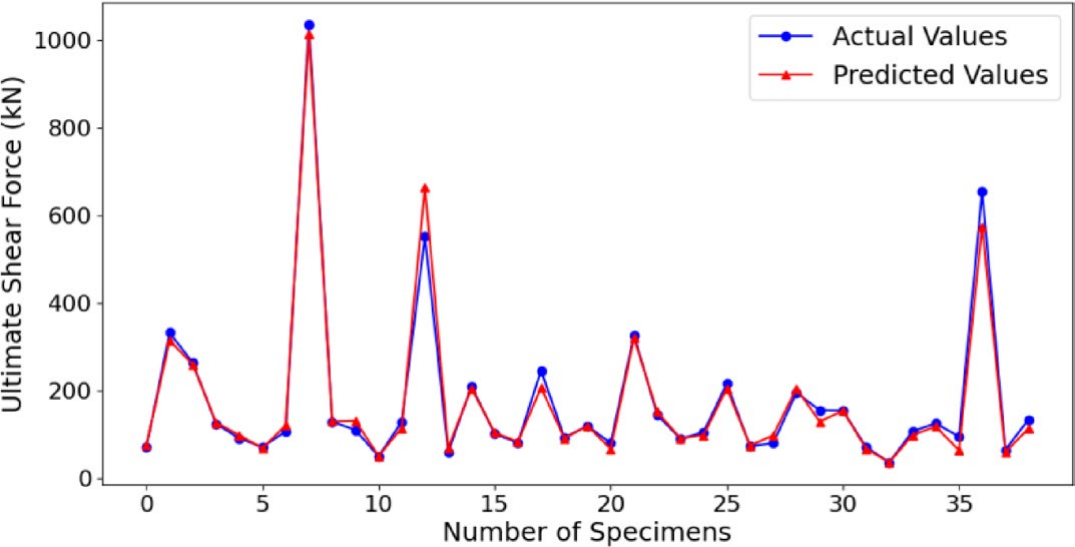
Comparison of actual and predicted shear strength values for Levy-DT algorithm.

Figure 7 provides a detailed comparison between the actual and predicted shear strength values for a series of specimens, as derived from the Levy-DT algorithm. The blue line in the figure represents the real shear strength measurements, while the red line illustrates the predicted values generated by the Levy-DT model. The close spacing of these two lines in most specimens indicates the close prediction of shear strength by the Levy-DT algorithm. The model captures rather appreciable, some important variations in shear strength over the dataset, hence making a valid performance comparison of RC beams under different conditions. One of the most striking features of the plot is the marked peaks and troughs in both observed and predicted values which reflect the inherent variability in the dataset owing to differing sample properties. The algorithm demonstrates an extremely high level of accuracy in peak matching where there is a significant increase in shear strength value and valley matching where there is a decrease in value. This serves as an indicator that the Levy-DT algorithm considerably recognizes and responds to those underlying structural patterns within shear strength, which exhibit pronounced variability across the samples. The strength of the algorithm is shown by how it works with all types of data points. Even in places where the shear strength has fast changes, the predicted numbers are close to the actual measurements. This indicates the model’s ability to generalize effectively across a diverse set of specimens, which might have different shapes or materials that change their shear behavior. The Levy-DT algorithm proves its trustworthiness in finding shear strength by keeping a near-perfect match between expected and real values; especially in complex datasets with many parameters. Another key point shown in Fig. 7 is how well the model deals with odd specimens. Even if small differences can be seen between guessed and true values, especially for some peaks, these errors are tiny and do not harm the overall quality of the model’s work. This indicates that the Levy-DT algorithm retains high predictive accuracy even in instances where challenge or odd conditions might be represented by data points. The small amount of error at these points indicates that the model can well handle noise or outliers in the data.

**Fig. 8:**
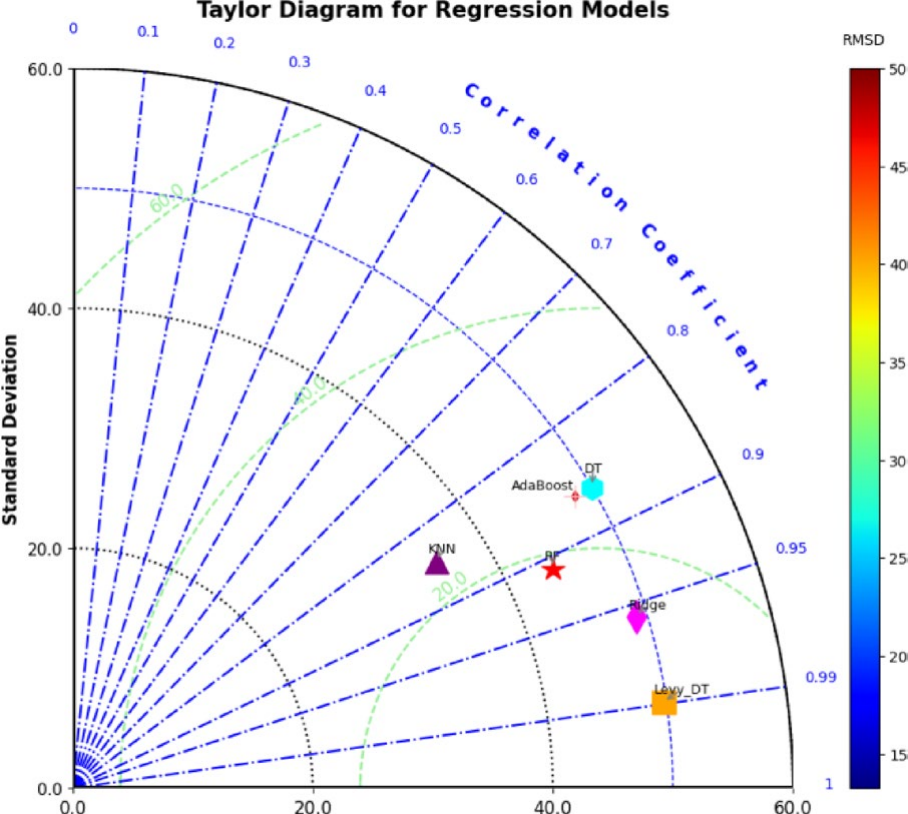
Taylor diagram representation of algorithm performance.

Figure 8 shows the Taylor diagram for the comparison of various ML models used to forecast the shear strength of RC T-beams. This diagram gives a clear idea of how model predictions match up with real values by looking at three important performance measures: standard deviation, correlation coefficient, and root mean square difference (RMSD). Each model is shown on the basis of these measures, helping to compare its predictive accuracy fully. The radial distance from the origin shows͏ the standard deviation of the predicted values while models nearer to the origin reflect a standard deviation like that of the observed data. The correlation coefficient is represented by circular lines that spread out from the center, with numbers going from 0 to 1. Models that show a stronger correlation are found near the right side, meaning there is a bigger relationship between what is predicted and what actually happens. The RMSD is shown with colors, and the gradient scale on the right side of the picture goes from 15 to 50. Models with lower RMSD values which are better are shown in cooler colors like blue and cyan while higher RMSD values are shown in warmer colors like orange and red. As seen in the picture, the Levy-DT algorithm shows the best results. It has a strong correlation (near 0.99), a pretty low standard deviation, and one of the lowest RMSD values shown in a cool blue color. This means Levy-DT does well in finding differences and is closely linked with real numbers, making it the best model out of the ones looked at. The traditional DT method is also near͏ly matched with Levy-DT, having a high correlation number and a bit higher standard deviation, though it still does alright compared to other models. KNN, on the other hand, sits farther from the perfect spot on the graph. It shows a lower correlation number (around 0.7), showing a weaker guessing ability. Its standard deviation is much higher than the actual values, and its RMSD goes into the warmer color range, pointing out its less precise performance. This implies that KNN has a hard time picking up the changes in the data and exhibits significantly higher prediction errors. The AdaBoost and RF models demonstrate satisfactory performance. Both are placed nearer to the center of the diagram than KNN, with correlation coefficients of around 0.8. However, their RMSD values are higher than those of Levy-DT and DT, which implies a trade-off between prediction accuracy and reduction of error. AdaBoost and RF also have a higher standard deviation than the actual data, so these models probably overstate or understate the variability of the dataset. Ridge regression lies between tree-based models and KNN; performance is moderate. It has a slightly higher correlation coefficient than AdaBoost and RF but lower than Levy-DT and traditional DT. The standard deviation is close to what is observed, and its RMSD is moderate, represented by a mid-range color on the scale. This shows that Ridge can deliver acceptable predictive performance but probably not as much as tree-based algorithms in reducing error. The Taylor diagram presented in Fig. 8 provides a clear and informative depiction of the differences in the performance of the ML models. Levy-DT comes out as the best model, showing better results in all key numbers, with DT not far behind. The better tree-based methods for finding shear strength emphasize this by the poor results of models like KNN, AdaBoost, and RF when it comes to correlation and standard deviation. The figure highlights the need for choosing models that find a good middle point between variance, correlation, and less error to make the right predictions in structural engineering.

## SHAP analysis of model interpretability

**Fig. 9:**
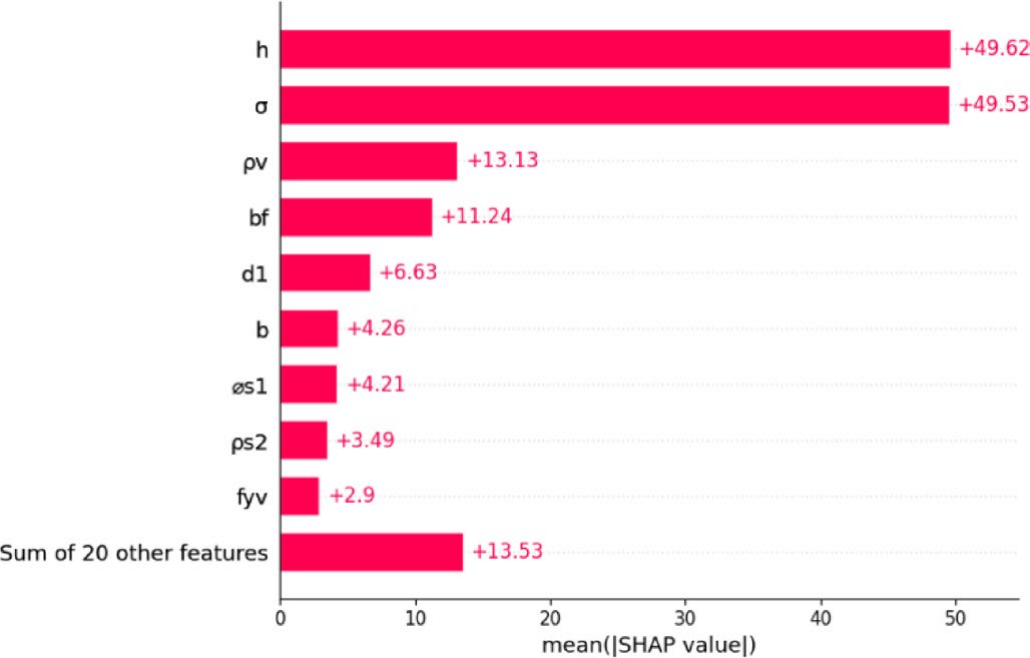
SHAP analysis of the contribution of features to the prediction of shear capacity of the T-beam.

In this section, SHAP analysis is used to make clear the ML model interpretation that is created to forecast the shear strength of RC T-beams. The SHAP framework gives a detailed view of the role of features that help to determine which variables exert the most significant impact on model predictions. Since it measures the effect of every feature, the mechanism provides a way to better understand the basic ways of working of the model and helps to make the decision process clearer. The SHAP result not only boosts the validity of what the model says but also guides structural engineering by showing key factors that influence shear capacity. Figure 9 presents the mean SHAP values of the features influencing the predicted shear strength (*Vu*) of T-beams. To enhance the physical interpretability and generalizability of the feature importance analysis, the axial load variable (*Pu*) is reformulated as a normalized parameter, *σ = Pu / A*, where A represents the gross cross-sectional area of the beam. To ensure consistent evaluation across specimens of varying sizes, the axial force is normalized into stress form. This transformation prevents raw force values from disproportionately influencing the multivariate analysis, which is a standard consideration in structural modeling. Representing axial load as stress offers a clearer depiction of internal mechanical demand within the section, rather than emphasizing its absolute scale. This approach also reduces potential overlap with size-related variables such as height and width. Moreover, the revised input structure enhances the clarity of feature attribution within the SHAP interpretability framework, without compromising the predictive accuracy of the model.

The results of the SHAP values in Fig. 9 indicate that the most influential features in the model’s prediction of shear strength are the overall beam height (*h*) and the normalized axial stress (*σ*), each exhibiting a mean SHAP value of approximately + 49. These variables jointly reflect the geometric scale and stress intensity within the cross-section, which are critical determinants of internal force distribution and failure mechanisms in RC T-beams. While *σ* effectively captures the mechanical impact of axial loading independent of beam size, h governs the lever arm and shear-resisting depth, both of which are foundational in shear design formulations. Beyond these dominant variables, the stirrup ratio (*ρ*_*v*_), flange width (*b*_*f*_), and effective depth (*d*_*1*_) also exhibit substantial contributions, with mean SHAP values ranging between approximately + 6 and + 13. These features are structurally meaningful, as they influence shear resistance through mechanisms such as transverse reinforcement effectiveness, flange engagement, and internal lever arm formation. Other notable contributors include web width (*b*), longitudinal reinforcement diameter (*ø*_*s*1_), and flange reinforcement ratio (*ρ*_*s*2_), each reflecting the detailed reinforcement configuration and geometry of the beam. Although their individual contributions are comparatively moderate, they collectively shape the internal stress field and crack propagation patterns. The yield strength of stirrups (*f*_*yv*_) also appears among the top variables, underscoring the mechanical role of transverse steel in resisting diagonal tension failures. The cumulative SHAP value of the remaining 20 features is approximately + 13.5, indicating that while many inputs are used by the model, a small subset of parameters predominantly governs the predictive behavior. This outcome confirms that the Levy-DT algorithm not only captures the multifaceted interactions governing shear strength but does so in a physically consistent and interpretable manner.

**Fig. 10:**
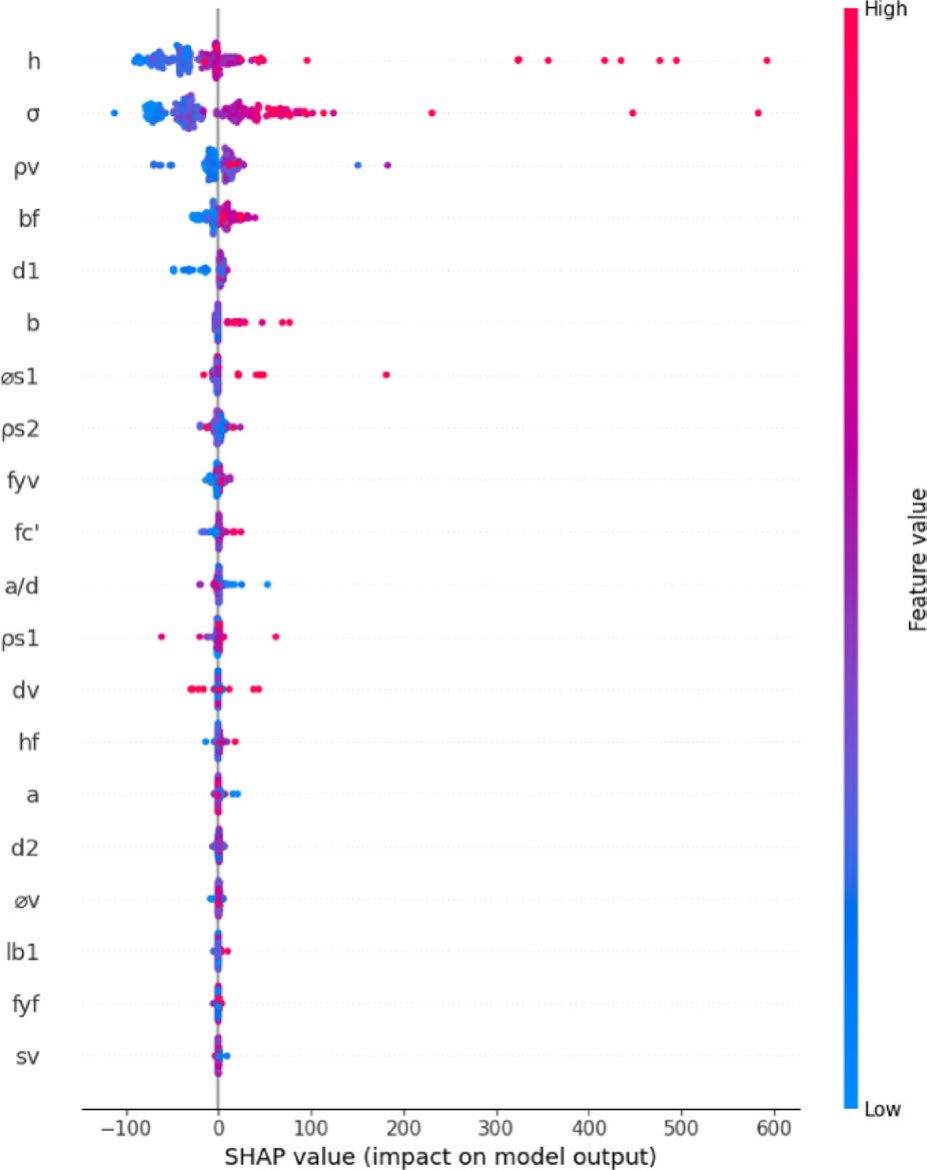
Analysis of SHAP values for evaluating feature contributions to the prediction of shear strength of the beam.

Figure 10 presents the SHAP summary plot, which visualizes how each input variable contributes to the model’s predictions of shear strength (*V*_*u*_) for individual T-beam samples. Each dot represents a SHAP value for a single prediction, where the x-axis shows the magnitude and direction of that feature’s effect on the output, and the color indicates the actual feature value (ranging from low in blue to high in red). Among all features, the overall beam height and the normalized axial stress emerge as the most influential, displaying the widest range of SHAP values. This highlights their dominant role in shaping model predictions. As discussed in relation to Fig. 9, *σ* reflects the mechanical impact of axial loading, capturing stress intensity independently of size, while *h* governs the internal lever arm and shear-resisting depth key structural parameters influencing shear behavior. The stirrup ratio, flange width, and effective depth also show notable SHAP impacts. These features directly contribute to shear resistance through mechanisms such as transverse reinforcement action, flange engagement, and internal force distribution. Additional contributing parameters include web width, longitudinal reinforcement diameter, flange reinforcement ratio, and stirrup yield strength each reflecting different aspects of reinforcement detailing and cross-sectional configuration. Other variables, such as concrete compressive strength, shear span-to-depth ratio, and shear depth, display moderate SHAP dispersion, indicating their structural relevance, although to a lesser degree in this dataset. Conversely, parameters such as flange depth, reinforcement cover depth, stirrup diameter, and loading area width exhibit low SHAP values across most samples, suggesting a limited role in the model’s decision-making process, likely due to narrow variability or secondary influence.

**Table 5:** Properties of a specific T-beam sample.

Parameter	Value		Parameter	Value	Parameter	Value
a/d	3.50		a	1050	f_yf_	360
b	150		l_b1_	90	a_g_	20
b_f_	300		l_b2_	90	f_c_’	35
h_f_	75		ρ_s1_	4.36	ρ_v_	0.34
d_1_	300		⌀_s1_	25	⌀_v_	6
d_v_	266		n_s1_	4	n_v_	2
d_2_	325		f_y_	400	s_v_	110
h	350		ρ_s2_	0.32	f_yv_	300
L	3000		⌀_s2_	10	σ	5.79
a_f_	900		N_s2_	2	V_u_	185

**Fig. 11:**
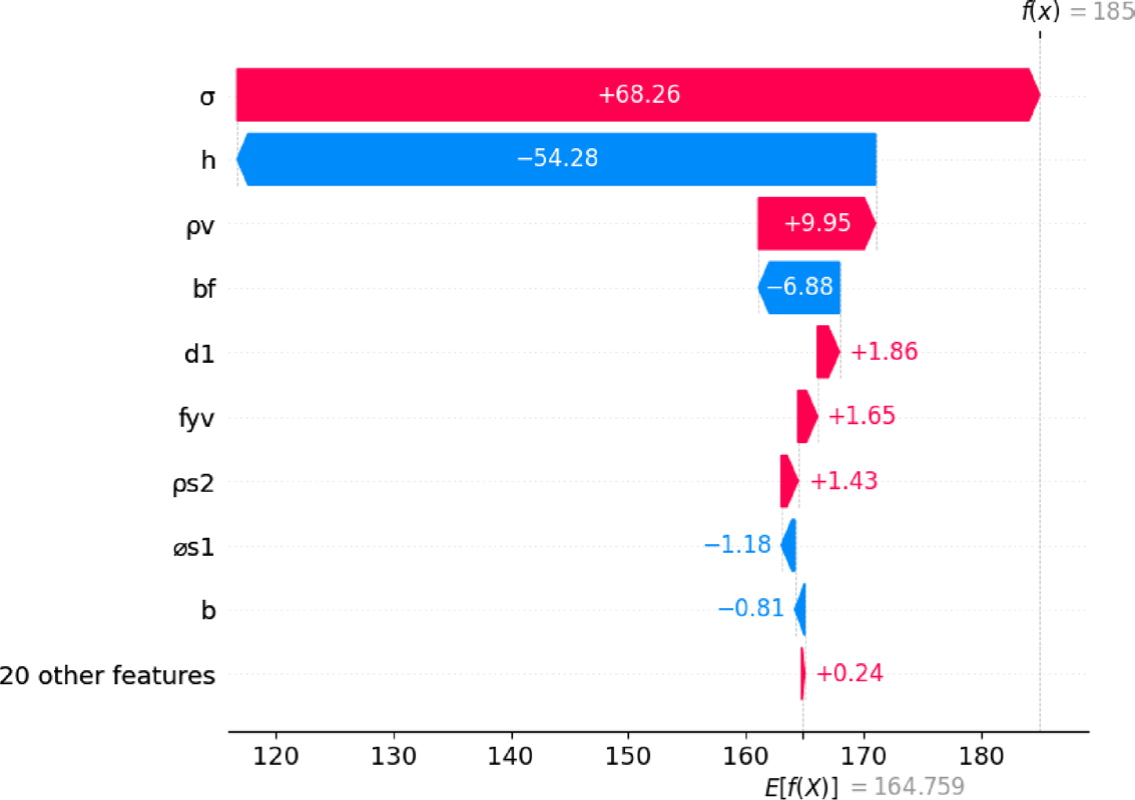
Shap waterfall plot detailing contributions of features to predicted output for a specific sample.

Figure 11 illustrates the SHAP waterfall plot for a representative prediction generated by the model, demonstrating how individual features influence the estimated shear strength for a specific T-beam instance, whose properties are shown in Table 5. The plot begins at the base value of the model, approximately 164.76, which represents the mean prediction in the dataset, and sequentially adds or subtracts the SHAP contributions of individual input features, resulting in a final predicted value of 185. The most dominant contributor is the normalized axial stress, which increases the prediction by + 68.26, highlighting the significant role of axial loading in enhancing shear capacity likely through mechanisms such as increased confinement and compressive stress redistribution. In contrast, the overall beam height exerts the most pronounced negative impact (–54.28), reflecting its inverse relationship with shear demand, where increased height may reduce the efficiency of shear-resisting mechanisms due to altered internal force paths or slenderness effects. The stirrup ratio (*ρ*_*v*_) also contributes positively (+ 9.95), aligning with its expected role in resisting diagonal shear through transverse reinforcement. Meanwhile, flange width (*b*_*f*_) shows a moderate negative contribution (–6.88), which could reflect variations in load distribution or local flange behavior. Other meaningful contributors include effective depth (*d*_1_), stirrup yield strength (*f*_*yv*_), and flange reinforcement ratio (*ρ*_*s*2_), each exerting smaller positive influences (between + 1.43 and + 1.86), consistent with their roles in forming the internal lever arm and improving transverse steel effectiveness. Features such as longitudinal reinforcement diameter and web width make minor negative adjustments (–1.18 and − 0.81, respectively), indicating limited but structurally interpretable effects on shear capacity. The combined impact of 20 other features not individually shown is minimal, suggesting that the model relies primarily on a core group of input variables for this particular prediction. Overall, the SHAP analyses demonstrate that the Levy-DT model not only reflects the physical behavior of the structural system but also offers interpretable insights into the importance of each input variable, thereby enabling more transparent and informed engineering assessments.

## Conclusions

This paper presents a comparative analysis of ML models to predict the mechanical properties of RC T-beams, emphasizing the Levy-DT algorithm. The original DT algorithm, though very efficient in a wide range of applications, harbors some inherent limitations. These include the propensity to overfit data and a limited ability to model highly complex or nonlinear relationships. All of these limitations bring a constraint on the generalization capability and, therefore, lower prediction accuracy in some contexts. The LF mechanism adds to the improvement of the DT algorithm by balancing more exploration and exploitation during model optimization. By integrating the Levy-DT model with the stability characteristics of LF, convergence rates are enhanced, and predictions become more accurate. This modification implies that, henceforth, the algorithm will be much better at escaping local minima and addressing generalization challenges that persist even after conventional hyperparameter optimization of the standard DT. The Levy-DT model outshines all the other ML models used in this study RF, AdaBoost, KNN, Ridge Regression, and optimized standard DT by delivering superior performance and achieving the highest R² value of 0.982. Such accuracy asserts Levy-DT’s capacity to deal with complex nonlinear issues in structural materials, wherein the accurate prediction of mechanical properties is crucial. Notably, this superior performance is achieved through comparison with an optimized baseline DT model that underwent systematic hyperparameter tuning, ensuring that the observed improvements represent genuine algorithmic enhancements rather than artifacts of unfair model comparison. In addition to its superior predictive performance, the Levy-DT algorithm demonstrated consistent robustness across training, testing, and cross-validation phases, confirming its strong generalization ability. The integration of the LF mechanism not only improved the model’s capacity to escape local optima but also contributed to more stable convergence behavior across multiple iterations. The incorporation of the LF mechanism not only enhanced the model’s ability to avoid local optima but also led to stable convergence across repeated experiments. These advantages are particularly evident in the convergence trends and sensitivity analyses, which demonstrated a consistent balance between computational efficiency and predictive precision under optimal conditions. Moreover, the interpretability of the proposed model is extensively analyzed through SHAP values. These results support both engineering intuition and previous empirical findings in the literature, affirming the model’s ability to capture meaningful relationships in the dataset. Importantly, the interpretability provided by SHAP values enhances the transparency of the model’s decision-making process, which is a critical consideration for the adoption of ML tools in structural design practices. Overall, this research presents an innovative ML-based hybrid strategy that delivers a robust and interpretable solution for estimating the shear capacity of RC T-beams. The model’s high predictive accuracy and explainability demonstrate the practical value of combining algorithmic optimization with transparent inference mechanisms in structural applications. This approach contributes meaningfully to data-driven structural engineering, where performance and interpretability are both essential. Nonetheless, the current analysis remains limited to RC T-beams, which suggests the need for future studies to evaluate the model’s adaptability across a broader range of structural elements. Additionally, integrating the Levy-enhanced decision tree framework with complementary ML methods may further enhance generalizability and predictive strength in diverse contexts. This method can offer richer and more reliable predictive insights by leveraging the strengths of diverse learning strategies.

## Data Availability

The data of this paper will be used in future studies, and it is only available upon individual requests to the corresponding author.The automated T-beam shear strength prediction ML application developed in this study is publicly accessible through the following link: [https://drive.google.com/file/d/1PrbBTs8E3JdW8ITuAUy5wwG2XWAE4SiR/view? usp=drive_link].
